# The Physical Activity Patterns among Rural Chinese Adults: Data from China National Nutrition and Health Survey in 2010–2012

**DOI:** 10.3390/ijerph15050941

**Published:** 2018-05-09

**Authors:** Caicui Ding, Chao Song, Fan Yuan, Yan Zhang, Ganyu Feng, Zheng Chen, Ailing Liu

**Affiliations:** Department of Nutrition and Health Education, National Institute for Nutrition and Health, Chinese Center for Disease Control and Prevention, Beijing 100050, China; dingcc@ninh.chinacdc.cn (C.D.); songchao@ninh.chinacdc.cn (C.S.); yuanfan@ninh.chinacdc.cn (F.Y.); zhangyan@ninh.chinacdc.cn (Y.Z.); fenggy@ninh.chinacdc.cn (G.F.); chenzheng@ninh.chinacdc.cn (Z.C.)

**Keywords:** physical activity, rural adults, China, CNNHS

## Abstract

China has experienced a rapid increase in non-communicable diseases (NCDs), especially in rural China. In addition to a dramatic increase in energy intake, the decrease in physical activity (PA) may be a reason. The study described the patterns and factors of physical activity and sedentary behaviors of 70,038 adults in rural China, based on data from the Chinese Nutrition and Health Survey (CNNHS) in 2010–2012. The mean working time of the employed subjects was 6.04 ± 1.3 day/week, 8.38 ± 2.2 h/day, of which 3.37 ± 2.8 h was sitting time. The occupational PA intensity was significantly relevant with occupation. The average transportation time of the rural Chinese adults was 57.9 ± 45.4 min, and 60.0% took the active transportation mode. The leisure-time PA (LTPA) participation rate was 3.8%, the LTPA time of those who had LTPA was 234.9 ± 231.3 min/week, lower participation was found in farmers and those in younger age groups, and those with lower educational and economic levels. The average domestic PA time, sedentary leisure-time, and sleeping time was 93.0 ± 72.7 min, 2.5 ± 1.4 h, and 7.9 ± 1.1 h, respectively. PA programs should be implemented in rural China, and the PA guidelines for farmers should be developed separately for the farming and non-farming seasons.

## 1. Introduction

Regular moderate intensity physical activity (PA) has significant benefits for health. It reduces the risk of a large number of diseases and conditions, prevents excessive weight gain, reduces the risk of falls and many cancers, and for the individuals who have chronic disease, it can reduce the risk of progression of their condition. Moreover, regular PA can help people sleep better, feel better, and perform daily tasks more easily [1].

Insufficient PA can cause a 20% to 30% increased risk of death, and adds to the burden of non-communicable diseases (NCDs) [2]. According to a study using data from 142 countries, physical inactivity cost health-care systems international $ (INT$) 53.8 billion, while related deaths contributed to $13.7 billion in productivity losses, and were responsible for 13.4 million Disability Adjusted of Life Years (DALYs) worldwide in 2013. The low-income and middle-income countries in the study had a larger proportion of the disease burden [3]. However, insufficient physical activity is on the rise in many countries [4,5,6].

By the end of 2016, the total population in China had reached about 1.38 billion, 42.7% of which was rural [7]. Modernization and urbanization have led to lifestyle changes and increasing risks for chronic diseases in China, especially in rural areas. From 2002 to 2012, the overweight and obesity rates of adults increased by a rate of 44.0% (from 26.6% to 38.3%) in rural China, significantly higher than the increase in urban areas (20.3%, from 37.9% to 45.6%). The increase rate of other chronic diseases among rural residents was also higher than that of the urban population. For example, the prevalence of hypercholesterolemia and type 2 diabetes increased by 304.2% (from 2.4% to 9.7%) and 366.7% (from 1.8% to 8.4%), respectively, in rural areas, while it increased by 197.6% (from 4.1% to 12.2%) and 173.3% (from 4.5% to 12.3%), respectively, in urban areas [8]. These more rapid increases in chronic diseases were partly because of the rapid transition of work and lifestyles in Chinese rural areas. It is very important to understand the situations of the risk factors and implement interventions to slow down the increase rate. In addition to a dramatic increase in energy intake from animal-source food and edible oils in the rural areas [9], the decrease in PA may be a reason. There were few national studies on the PA patterns of adults in rural China. There were only some studies on PA in parts of the country [10,11,12].

The current study is based on data from the Chinese Nutrition and Health Survey (CNNHS) in 2010–2012, the largest and most comprehensive study of nutrition and health outcomes ever conducted in China. The objectives of the study were to describe patterns of physical activity and sedentary behaviors among rural Chinese adults and explore the factors associated with physical activity in rural areas, which can help to develop valid intervention strategies and provide useful information for other countries in the same situation as China.

## 2. Materials and Methods

### 2.1. Study Design

The CNNHS was a nationally representative cross-sectional study conducted by the National Institute for Nutrition and Health, Chinese Center for Disease Control and Prevention (NINH, China CDC) to assess the health and nutrition of Chinese civilians. The 2010–2012 survey covered all 31 provinces, autonomous regions, and municipalities directly under the central government throughout China (except for Taiwan, Hong Kong, and Macao). The country was divided into four strata: large cities, small- and medium-sized cities, general rural areas, and poor rural areas, according to their characteristics of economy and social development, using data from the China National Bureau of Statistics, in which cities were divided mainly by population size and gross national product, and the list of poor rural areas published by the State Council of China [13]. The poor rural areas referred to the areas where the income of residents was below 625 Yuan per capita per year, according to the definition in the National Program for Rural Poverty Alleviation and Development (2001–2010) [14]. General rural areas referred to the other rural counties, except for the poor rural areas.

Participants were recruited using a stratified multistage cluster and probability proportional to size (PPS) sampling design. The sampling method was reported in a previous study [15]. Ethics approval was obtained from the Ethics Committee of NINH, China CDC (2013–2018). All participants provided written informed consent for their participation in the survey.

### 2.2. Participants and Setting

The current study focused on rural Chinese adults. The rural participants were defined as residents with a rural record in the Chinese household registration. The study included the participants in both ‘general rural areas’ and ‘poor rural areas’, and those with rural household records in ‘small- and medium-sized cities’ were also included. The number of respondents to the Rural Physical Activity Questionnaire was 71,569. The participants who had missing responses for the measured activities (*n* = 1102) and the demographic (sex, age, area, employment status) data (*n* = 429) were excluded. The final analytical sample included 70,038 (97.9%) rural Chinese adults.

### 2.3. Data Collection

Information on the physical activity of participants was collected by trained investigators using face-to-face interviews. The rural participants were asked to complete the Rural Questionnaires, which included questions about time and intensity of work, transportation, domestic and leisure-time physical activity, time of leisure-time sedentary behaviors, and sleeping.

Participants reported their employment status as employed, farmer, or unemployed. The employed included those who were employed in non-farming occupations and self-employed shop keepers. The unemployed did neither farming nor employed work, and their main activity was housework. Occupational PA data were only collected among those who reported being employed or farmers.

### 2.4. Physical Activity and Sedentary Behaviors Assessment

The study assessed physical activity across a comprehensive set of domains, including occupational PA (OPA), transportation PA (TPA), leisure-time PA (LTPA), and domestic PA (DPA). OPA intensity was divided into three levels according to the level of efforts and sitting or standing time during work: light OPA (i.e., clerk, shop assistant, and farming of a light effort), moderate OPA (i.e., truck driver, electrician, and farming of a moderate effort), and vigorous OPA (i.e., miner, porter, and farming of a vigorous effort). Activity patterns of farmers are seasonal. Farmers do moderate-to-vigorous-intensity, long-hour planting, and harvesting activities during the farming season, and less-intense field maintenance during the non-farming season. To capture the seasonality of activities, those in the farmer subsample were asked to estimate the length of the farming season and non-farming season every year, and to recall the OPA separately during the farming and non-farming seasons. In accordance with the contribution to health, the transportation modes were divided into active and inactive transportation. The active transportation included walking and bicycling, while the inactive transportation included taking a bus and riding in a car/truck. LTPA was defined as any physical activity for the purpose of recreation and/or fitness, such as leisure walking, climbing, playing balls, martial arts, and so on. Domestic PA was defined as any housework that involves physical activity, such as cleaning and maintaining the house, cooking, caring for family, and so on. Leisure-time sedentary behaviors included lying down, sitting (reading, or using the computer and other forms of screen-based entertainment), watching TV, and playing cards. The leisure-time sedentary time of more than 2 h/day was associated with the risk of chronic diseases and all-cause mortality [16], thus the proportion of leisure-time sedentary time of more than 2 h/day was calculated along with the average sedentary time. Sleeping time was also investigated in the questionnaire. Both insufficient sleep and too much sleep were health risk factors, and 7 to 9 h/day sleeping time was considered appropriate for adults [17,18]. Thus, the proportion of less than 7 h/day and more than 9 h/day was calculated along with the average sleeping time.

### 2.5. Statistical Analysis

The participants were divided into sub-classes according to socioeconomic factors: occupation, gender, age, marital status, education level, and family’s economic level. *t*-tests, variance analysis, and nonparametric tests were performed for analysis of physical activity and sedentary behaviors among groups. Intensity of OPA was divided into “moderate or vigorous” OPA (MVOPA) and “light” OPA according to their contribution to total level of PA. Participation in LTPA was dichotomized as “none” and “any” because of a large proportion of zero values (96.2%). Chi-square tests examined bivariate associations between independent variables and dependent variables (LTPA). The statistical software package SAS version 9.4 (SAS Institute Inc., Cary, NC, USA) was used for data analysis. Using two-sided tests, the significance level was set at *p* < 0.05.

## 3. Results

### 3.1. Characteristics of the Sample

The demographic characteristics among the subjects are presented in Table 1. Of the participants, 55.8% were female, the average age was 50.7 ± 14.5 years, 89.7% were married, 56.9% were illiterate and had graduated from primary school.

### 3.2. Occupational Physical Activity (OPA)

In the study sample, the mean working time of the employed subjects was 6.04 ± 1.3 day/week, 8.38 ± 2.2 h/day, of which 3.37 ± 2.8 h was sitting time. The average length of the farming season was 5.6 ± 2.4 months, the women’s mean farming season months were lower than that of the men (*p* < 0.01). The length of the farming season decreased as age and economic level increased (*p* < 0.01). The OPA time of the employed and farmers, stratified by population characteristics, are listed in Table 2.

The OPA intensity was significantly relevant to occupation. Farmers during the farming season had the highest OPA intensity, while farmers during the non-farming season had the lowest OPA intensity. The OPA intensity was also relevant with region types; the participants in poor rural areas had the highest OPA intensity. In each occupational group, the OPA intensity of men was higher than that of women (Table 3).

### 3.3. Transportation

The average transportation time of rural Chinese adults was 57.9 ± 45.4 min, which was higher in the farmers, the males, and in the poor rural areas. The transportation time gradually reduced with the increase in age, educational level, and economic level (Table 4).

Among the rural Chinese adults, 60.0% took the active transportation mode in the last three months. More women chose the active transportation mode than men, more residents in poor rural areas took the active transportation mode when compared with other regions. The percentage of subjects who took the active transportation mode gradually increased with age, but reduced with the increase in educational and economic levels. More employed subjects took the inactive transportation mode than farmers and the unemployed subjects (Table 4).

### 3.4. Leisure-Time Physical Activity (LTPA)

The LTPA participation rate of rural Chinese adults was 3.8%, higher participation in LTPA was found among those who were women, older, in small- and medium-sized cities, employed in non-farming occupations or not employed, or in higher educational or economic levels (Table 5).

The average LTPA time of rural Chinese adults who had LTPA was 234.9 ± 231.3 min/week. The LTPA time per week was longer among those who were female, those in the 45–59.9 years old age group, and the unemployed subjects. The LTPA time per week was the shortest among those in poor rural areas.

### 3.5. Domestic Physical Activity (DPA)

The average DPA time of rural Chinese adults was 93.0 ± 72.7 min. More time was found among those who were women, 45–59 years old, in poor rural areas, married, unemployed, or in lower educational or economic levels (Table 5).

### 3.6. Leisure-Time Sedentary Behaviors (LTSB)

Approximately 97% of the rural Chinese adults had LTSB, the average leisure-time sedentary time (LTST) was approximately 2.5 h. Those who were men, 18–44 years old, married, in higher educational levels, in higher economic levels, or unemployed had longer LTST. The subjects in poor rural areas had shorter LTST than those in the other two region types. In the study, 78.6% of the subjects had LTSB >2 h/day. A greater percentage of LTST ≥2 h/day was found among those who were men, in younger age groups, married, in a higher educational level, in a higher economic level, or unemployed (Table 6).

### 3.7. Sleeping Time

The average sleeping time of rural Chinese adults was approximately 7.9 h, and was higher among those who were women, 18–44 years old, in poor rural areas, unmarried, in a lower educational level, in a lower economic level, or unemployed. The percentage of sleeping time <7 h was 11.2% among rural Chinese adults. A greater percentage of sleeping time <7 h was found among those who were men, in older age groups, in small- and medium-sized cities, unmarried, in a lower educational level, or unemployed (Table 6).

## 4. Discussion

This study found that the LTPA participation rate of rural Chinese adults from 2010 to 2012 was only 3.8%, which was much lower than the objective of the National Fitness Program (30% prevalence of regular LTPA), which was consistent with findings from previous studies [12,19]. There was not a significantly improved in LTPA participation rate when compared with the results of the national nutrition surveys in 2002 [20], and the rate was lower than that of urban China [21]. In our study, subjects were more likely to participate in LTPA if they were in older age groups, in a higher educational level, in a higher economic level, or not farmers, which was similar to results from previous studies [22]. Compared with Brazil and other countries [23,24,25], the LTPA prevalence in adults was lower in rural China. One possible reason was that LTPA was not very common for rural Chinese adults. Thus, through the decline of work-related activity, many adults would lose a substantial amount of their overall physical activity.

This study found that the participation rate in MVOPA was lower than that of the survey in 2002 [20], which was explanatory. Urbanization and technological advances in the workplace have been reported to be associated with a significant proportion of the decline in physical activities, particularly occupational physical activities [26,27]. Additionally, it has been suggested that mechanization in the agricultural sector may lead to increased sedentary behaviors among farmers [28].

In the current study, the LTST of rural Chinese adults was higher than it was in 2002 [20], which was consistent with findings from other studies [12,22]. The social correlations of LTST, such as with men and younger adults, were similar when compared with other countries [29,30]. Sedentary time is viewed as an independent risk factor for adverse health, especially for heart disease, diabetes, and obesity [31]. Key populations should be a focus.

Among the farmers, the OPA intensity was highly related to the farming season, which was well understood and reported in previous studies [32]. The prevalence of LTPA among farmers was very low, and our study found that the LTPA prevalence in farmers was the lowest among the three occupational groups. One explanation could be that in the farming season, a lot of time was spent on highly intense physical activities, conducting diverse agricultural activities, and there was no time and body strength for farmers to have LTPA. Furthermore, in the non-farming season, farmers spent a lot of time on very low intensity physical activities, such as sleeping and resting, house management, and social activities, but did not develop the habit of exercise. Some studies had reported that there was a significant increase in Body Mass Index (BMI), body fat, and the hypertension prevalence rate in the non-farming season when compared with the farming season [32]. Thus, there is a need to develop physical activity guidelines for farmers separately for the farming season and non-farming season.

Among the three region types, participants in poor rural areas had the highest OPA intensity, the longest TPA time, the longest DPA time, the shortest sedentary time, and the longest sleeping time, but also had the lowest LTPA participation rate. These differences were likely due to the lower urbanization level in poor rural areas, including the utility of the modern farm machines, unpopular of the internet, computer and smart phones, and less social activities. Meanwhile, the residents may also know less about the benefits of physical activity.

China has been undergoing a rapid social and health transition in the past few decades. The morbidity and mortality rates of NCDs has increased rapidly, especially in rural China. On the one hand, the reason may be that the rural health service system was not very good; while on the other hand, it may lie in the rural residents’ low participation in healthy lifestyles, including an unreasonable diet structure and the lack of regular exercising [33]. This study suggested that in rural China, the prevalence of LTPA was low, the participation rate of MVOPA was decreasing, and the sedentary time was increasing. The same situations may also occur in countries with similar economic development. The findings in the current study are useful for other developing countries that are undergoing rapid work and lifestyle transitions. It is very important to understand the situations and implement suitable PA interventions at the key moment in order to prevent the rapid increase of chronic diseases.

The present study has a few limitations. First, the study did not investigate the daily working hours of farmers, so the physical activity level of the entire rural Chinese resident base was not available. Second, the study was a descriptive cross-sectional analysis. These results can only inform us of associations between potential predictive factors and physical activity prevalence.

## 5. Conclusions

This study suggested that in rural China, the prevalence of LTPA was low, the participation rate of MVOPA was decreasing, and the sedentary time was increasing. Physical activity programs should be implemented in rural China, especially in key populations, such as younger adults. The physical activity guidelines for farmers should be developed separately for the farming season and non-farming season.

## Figures and Tables

**Table 1:** Characteristics of the study subjects stratified by occupation.

Variables	Total	Employed	Farmers	Unemployed	*p*-Value
Total, *n* (%)	70,038 (100.0)	11,744 (16.7)	39,407 (52.3)	18,887 (27.0)	
Female, *n* (%)	39,098 (55.8)	4078 (34.7)	20,344 (51.6)	14,676 (77.7)	<0.001 *
Age (year), Mean ± SD	50.7 ± 14.5	43.4 ± 12.2	50.5 ± 12.8	55.8 ± 16.9	<0.001 ^#^
Age group (year), *n* (%)					<0.001 *
18–44.9	24,452 (34.9)	6498 (55.3)	13,060 (33.1)	4894 (25.9)	
45–59.9	26,231 (37.5)	4192 (35.7)	16,659 (42.3)	5380 (28.5)	
≥60	19,355 (27.6)	1054 (9.0)	9688 (24.6)	8613 (45.6)	
Region type, *n* (%)					<0.001 *
Small and medium-sized cities	11,126 (15.9)	3115 (26.5)	4530 (11.4)	3481 (18.4)	
General rural areas	35,349 (50.5)	6277 (53.4)	18,976 (48.2)	10,096 (53.5)	
Poor rural areas	23,563 (33.6)	2352 (20.1)	15,901 (40.4)	5310 (28.1)	
Married, *n* (%)	62,794 (89.7)	10,743 (91.5)	36,287 (92.1)	15,764 (83.5)	<0.001 *
Middle school graduate or higher, *n* (%)	30,186 (43.1)	7986 (68.0)	15,737 (39.9)	6463 (34.2)	<0.001 *
Family’s economic level (Yuan/Year/per capita), *n* (%)					<0.001 *
<5000	25,782 (36.8)	2519 (21.4)	15,892 (40.3)	7371 (39.0)	
5000–9999	20,194 (28.8)	3333 (28.4)	11,765 (29.9)	5096 (27.0)	
≥10,000	21,484 (30.7)	5403 (46.0)	10,442 (26.5)	5639 (29.9)	
unknown	2578 (3.7)	489 (4.2)	1308 (3.3)	781 (4.1)	

* *p* < 0.001 was calculated by Chi-square tests; ^#^ *p* < 0.001 was calculated by variance analysis.

**Table 2:** Occupational physical activity time of the employed and farmers.

Variables	The Employed						Farmers	
	Working Days/Week (Mean ± SD)	*p*-Value	Working Hours/Day (Mean ± SD)	*p*-Value	Sitting Hours during Work (Mean ± SD)	*p*-Value	Farming Season Months (Mean ± SD)	*p*-Value
Sex		0.012 *		<0.001 *		<0.001 *		<0.001 *
Female	6.1 ± 1.3		8.2 ± 2.2		3.9 ± 2.9		5.5 ± 2.4	
Male	6.0 ± 1.3		8.5 ± 2.1		3.1 ± 2.8		5.6 ± 2.4	
Age group (year)		0.012 ^#^		<0.001 ^#^		<0.001 ^#^		<0.001 ^#^
18–44.9	6.0 ± 1.2		8.5 ± 2.0		3.5 ± 2.9		5.8 ± 2.3	
45–59.9	6.1 ± 1.3		8.3 ± 2.2		3.1 ± 2.7		5.6 ± 2.4	
≥60	5.9 ± 1.5		7.7 ± 2.6		3.3 ± 2.9		5.2 ± 2.4	
Region type		<0.001 ^#^		0.057 ^#^		0.032 ^#^		<0.001 ^#^
Small- and medium-sized cities	6.1 ± 1.3		8.4 ± 2.3		3.5 ± 3.0		6.2 ± 2.5	
General rural areas	6.0 ± 1.3		8.3 ± 2.0		3.4 ± 2.8		5.2 ± 2.4	
Poor rural areas	6.0 ± 1.3		8.5 ± 2.2		3.2 ± 2.7		5.8 ± 2.2	
Marital Status		0.001 *		0.024 *		0.883 *		0.133 *
Yes	6.1 ± 1.3		8.4 ± 2.2		3.4 ± 2.8		5.6 ± 2.4	
No	5.9 ± 1.3		8.2 ± 2.1		3.4 ± 2.8		5.5 ± 2.3	
Educational Level		0.003 *		<0.001 *		<0.001 *		0.817 *
Illiterate and primary school graduate	6.0 ± 1.4		8.2 ± 2.3		3.1 ± 2.8		5.6 ± 2.3	
Middle school graduate or higher	6.1 ± 1.2		8.4 ± 2.1		3.5 ± 2.9		5.6 ± 2.4	
Family’s economic level (Yuan/Year/per capita)		0.001 ^#^		<0.001 ^#^		<0.001 ^#^		<0.001 ^#^
<5000	6.0 ± 1.3		8.5 ± 2.2		3.1 ± 2.8		5.7 ± 2.4	
5000–9999	6.0 ± 1.3		8.3 ± 2.1		3.2 ± 2.8		5.6 ± 2.4	
≥10,000	6.1 ± 1.2		8.4 ± 2.2		3.6 ± 2.9		5.4 ± 2.4	
Total	6.0 ± 1.3		8.4 ± 2.2		3.4 ± 2.8		5.6 ± 2.4	

* *t*-tests; ^#^ Nonparametric Tests: Independent Samples.

**Table 3:** Occupational physical activity intensity of adults stratified by occupation in rural China (%).

Variables	The Employed			Farmers					
				During Farming Season			During Non-Farming Season		
	Light	Moderate	Vigorous	Light	Moderate	Vigorous	Light	Moderate	Vigorous
Sex									
Female	69.1	26.3	4.6	11.7	59.6	28.6	67.1	30.6	2.3
Male	37.5	43.4	19.0	7.6	54.7	37.6	56.7	39.3	4.0
Age group (year)									
18–44.9	48.9	38.4	12.7	8.0	54.9	37.2	59.2	36.5	4.3
45–59.9	45.7	37.7	16.7	8.5	58.6	32.9	62.4	34.7	2.9
≥60	57.4	31.0	11.6	14.3	58.3	27.4	65.2	33.0	1.9
Region type									
Small- and medium-sized cities	52.3	37.1	10.6	11.7	55.5	32.8	63.2	34.2	2.6
General rural areas	48.3	37.5	14.2	10.1	67.2	22.7	68.1	29.5	2.4
Poor rural areas	44.1	38.1	17.9	8.8	45.9	45.3	54.5	41.5	4.1
Marital Status									
Yes	48.2	37.4	14.4	9.5	57.6	32.9	62.2	34.8	3.1
No	51.4	38.7	9.9	12.4	53.4	34.2	60.4	35.8	3.8
Educational Level									
Illiterate and primary school graduate	44.1	38.7	17.1	9.4	56.7	33.9	61.6	35.2	3.2
Middle school graduate or higher	50.6	36.9	12.5	10.3	58.1	31.6	62.7	34.4	3.0
Family’s economic level									
<5000	42.6	37.8	19.7	10.6	53.9	35.5	60.4	36.6	3.1
5000–9999	45.7	40.1	14.2	8.9	58.4	32.7	63.1	33.8	3.1
≥10,000	52.7	36.2	11.1	9.3	59.8	30.9	63.3	33.4	3.3
Total	48.5	37.5	14.0	9.8	57.3	33.0	62.0	34.9	3.1

**Table 4:** Transportation time and mode stratified by social characteristics.

Variables	Transportation Time (min/day, Mean ± SD)	*p-*Value	Transportation Mode (%)			*p*-Value
			Active *	Inactive ^#^	No Transportation	
Sex		<0.001 ^a^				<0.001 ^c^
Female	55.3 ± 44.1		66.1	30.3	3.6	
Male	61.1 ± 46.8		52.4	45.6	2.0	
Age group (year)		<0.001 ^b^				<0.001 ^c^
18–44.9	59.3 ± 45.7		49.1	49.4	1.4	
45–59.9	58.7 ± 44.9		60.1	37.9	2.0	
≥60	54.9 ± 45.5		73.7	20.3	6.0	
Region type		<0.001 ^b^				<0.001 ^c^
Small- and medium-sized cities	55.1 ± 45.8		60.9	35.9	3.1	
General rural areas	52.5 ± 39.4		56.5	41.4	2.1	
Poor rural areas	67.2 ± 51.7		65.0	31.1	3.9	
Marital Status		<0.001 ^a^				<0.001 ^c^
Yes	58.2 ± 45.3		59.0	38.5	2.5	
No	54.6 ± 46.1		68.9	24.8	6.3	
Educational Level		<0.001 ^a^				<0.001 ^c^
Illiterate and primary school graduate	58.4 ± 46.3		67.7	28.3	3.9	
Middle school graduate or higher	57.1 ± 44.1		49.9	48.6	1.5	
Family’s economic level (Yuan/Year/per capita)		<0.001 ^b^				<0.001 ^c^
<5000	60.4 ± 47.7		68.6	27.9	3.6	
5000–9999	57.3 ± 44.2		58.7	38.6	2.7	
≥10,000	55.5 ± 43.6		51.1	46.5	2.4	
Occupation		<0.001 ^b^				<0.001 ^c^
Employed	51.0 ± 39.3		37.5	62.3	0.2	
Farmers	63.1 ± 46.8		63.4	35.4	1.2	
Unemployed	51.3 ± 44.5		67.1	24.9	8.0	
Total	57.9 ± 45.4		60.0	37.1	2.9	

^a^*t*-tests; ^b^ Nonparametric Tests: Independent Samples; ^c^ Chi-square tests; * Active transportation: walking or bicycling; ^#^ Inactive transportation: taking a bus or riding in a car/truck.

**Table 5:** The leisure-time physical activity (LTPA) * participation rate, LTPA time of those who had LTPA and domestic physical activity (PA) time of rural Chinese adults.

Variables	LTPA (%)	*p*-Value	LTPA Time of MV ^#^ Intensity (min/week, Mean ± SD)	*p*-Value	Domestic PA (min/day, Mean ± SD)	*p*-Value
Sex		0.048 ^a^		<0.001 ^b^		<0.001 ^b^
Female	3.9		252.8 ± 236.3		125.3 ± 68.5	
Male	3.6		210.5 ± 222.2		52.2 ± 55.3	
Age group (year)		<0.001 ^a^		<0.001 ^c^		<0.001 ^c^
18–44.9	3.0		206.6 ± 212.1		89.0 ± 71.0	
45–59.9	4.1		251.7 ± 234.1		98.5 ± 75.0	
≥60	4.4		237.7 ± 241.2		90.6 ± 71.2	
Region type		<0.001 ^a^		<0.001 ^c^		<0.001 ^c^
Small- and medium-sized cities	5.3		236.9 ± 241.2		91.5 ± 76.6	
General rural areas	4.3		242.8 ± 225.6		87.6 ± 70.0	
Poor rural areas	2.4		211.6 ± 235.0		101.8 ± 74.1	
Marital Status		0.124 ^a^		0.868 ^b^		<0.001 ^b^
Yes	3.8		235.2 ± 231.6		93.7 ± 73.1	
No	3.5		232.6 ± 229.2		86.8 ± 69.3	
Educational Level		<0.001 ^a^		0.155 ^b^		<0.001 ^b^
Illiterate and primary school graduate	3.1		241.8 ± 235.2		102.4 ± 73.9	
Middle school graduate or higher	4.7		229.0 ± 227.8		80.6 ± 69.1	
Family’s economic level		<0.001 ^a^		0.177 ^c^		<0.001 ^c^
<5000	3.4		237.1 ± 221.8		97.2 ± 73.9	
5000–9999	3.5		234.9 ± 240.9		93.3 ± 72.1	
≥10,000	4.6		233.8 ± 233.5		88.7 ± 72.3	
Occupation		<0.001 ^a^		<0.001 ^c^		<0.001 ^c^
Employed	5.4		204.8 ± 200.3		52.8 ± 55.0	
Farmers	2.0		228.1 ± 238.5		94.7 ± 70.3	
Unemployed	6.5		254.7 ± 239.5		114.5 ± 77.0	
Total	3.8		234.9 ± 231.3		93.0 ± 72.7	

* LTPA: Leisure-time physical activity.^.a^ Chi-squaretests; ^b^ t-tests; ^c^ Nonparametric Tests: Independent Samples; ^#^ MV: Moderate or vigorous.

**Table 6:** Time of sedentary behaviors and sleeping among rural Chinese adults.

Variables	Sedentary Behaviors (h/day, Mean ± SD)	*p*-Value	Sedentary Behaviors ≥2 h/day (%)	*p*-Value	Sleeping Time (h/day, Mean ± SD)	*p*-Value	Sleeping Time <7 h/day (%)	Sleeping Time ≥9 h/day (%)	*p*-Value
Sex		<0.001 ^b^		<0.001 ^a^		<0.001 ^b^			<0.001 ^a^
Female	2.4 ± 1.4		77.0		7.9 ± 1.2		10.9	23.0	
Male	2.5 ± 1.4		80.6		7.8 ± 1.1		11.5	19.8	
Age group (year)		<0.001 ^c^		<0.001 ^a^		<0.001 ^c^			<0.001 ^a^
18–44	2.5 ± 1.3		81.9		8.0 ± 1.0		6.4	23.6	
45–59	2.4 ± 1.4		78.5		7.8 ± 1.1		11.8	18.2	
≥60	2.4 ± 1.5		74.5		7.8 ± 1.3		16.4	23.8	
Region type		<0.001 ^c^		<0.001 ^a^		<0.001 ^c^			<0.001 ^a^
Small and medium-sized cities	2.6 ±1.5		78.8		7.8 ± 1.2		13.6	19.4	
General rural areas	2.5 ± 1.3		80.8		7.8 ± 1.1		11.7	20.0	
Poor rural areas	2.3 ±1.5		75.1		8.0 ± 1.1		9.3	25.2	
Marital Status		<0.001 ^b^		<0.001 ^a^		<0.001 ^b^			<0.001 ^a^
Yes	2.6 ± 1.6		78.7		7.8 ± 1.1		11.0	21.0	
No	2.4 ± 1.4		77.4		7.9 ± 1.3		12.9	27.0	
Educational Level		<0.001 ^b^		<0.001 ^a^		<0.001 ^b^			<0.001 ^a^
Illiterate and primary school graduate	2.4 ± 1.5		75.6		7.9 ± 1.2		12.1	23.9	
Middle school graduate or higher	2.5 ± 1.3		82.5		7.8 ± 1.1		10.0	18.7	
Family’s economic level		<0.001 ^c^		<0.001 ^a^		<0.001 ^c^			<0.001 ^a^
<5000	2.4 ± 1.4		75.3		7.9 ± 1.2		11.1	24.8	
5000–9999	2.5 ± 1.4		79.9		7.8 ± 1.1		10.5	20.7	
≥10,000	2.5 ± 1.4		80.9		7.8 ± 1.1		11.4	19.3	
Occupations		<0.001 ^c^		<0.001 ^a^		<0.001 ^c^			<0.001 ^a^
Employed	2.3 ± 1.3		76.9		7.8 ± 1.0		9.8	15.2	
Farmers	2.3 ± 1.2		78.4		7.8 ± 1.1		10.5	20.7	
Unemployed	2.8 ± 1.8		79.9		8.0 ± 1.3		13.4	27.6	
Total	2.5 ± 1.4		78.6		7.9 ± 1.1		11.2	21.6	

^a^ Chi-square tests; ^b^ *t*-tests; ^c^ Nonparametric Tests: Independent Samples.
